# Risk Management and Treatment of Coagulation Disorders Related to COVID-19 Infection

**DOI:** 10.3390/ijerph18031268

**Published:** 2021-01-31

**Authors:** Christian Zanza, Fabrizio Racca, Yaroslava Longhitano, Andrea Piccioni, Francesco Franceschi, Marco Artico, Ludovico Abenavoli, Aniello Maiese, Giovanna Passaro, Gianpietro Volonnino, Raffaele La Russa

**Affiliations:** 1 Department of Emergency Medicine, Gemelli, IRCCS (Scientific Institute for Hospitalization and Treatment), Catholic University of Rome-Teaching Hospital Foundation A, 00168 Rome, Italy; christian.zanza@live.it (C.Z.); andrea.piccioni@policlinicogemelli.it (A.P.); francesco.franceschi@unicatt.it (F.F.); 2 “Nuovo Ospedale Alba-Bra”, Foundation and Department of Anesthesia, Critical Care and Emergency Medicine, Pietro and Michele Ferrero Hospital, 12051 Verduno, Italy; 3 Department of Anesthesiology and Critical Care Medicine, Azienda Ospedaliera SS. Antonio e Biagio e Cesare Arrigo, 15121 Alessandria, Italy; fracca@ospedale.al.it (F.R.); lon.yaro@gmail.com (Y.L.); 4 Department of Sensory Organs, Sapienza University of Rome Policlinico Umberto I, 00155 Rome, Italy; marco.artico@uniroma1.it; 5 Department of Health Sciences, Magnae Grecia University of Catanzaro, 88100 Catanzaro, Italy; l.abenavoli@unicz.it; 6 Department of Surgical Pathology, Medical, Molecular and Critical Area, Institute of Legal Medicine, University of Pisa, 56126 Pisa, Italy; 7 Gemelli IRCCS Research Hospital, Fondazione Policlinico Universitario A, 00168 Rome, Italy; passaro.giovanna@gmail.com; 8 Department of Anatomy, Histology, Forensic Medicine and Orthopedics, Sapienza University, 00161 Rome, Italy; gianpietro.volonnino@uniroma1.it (G.V.); raffaele.larussa@uniroma1.it (R.L.R.)

**Keywords:** COVID-19, deep vein thrombosis, pulmonary embolism, coagulopathy, venous thromboembolism, thromboprophylaxis

## Abstract

Coronavirus disease 2019 (COVID-19) is an emerging infectious disease. Bilateral pneumonia, acute respiratory failure, systemic inflammation, endothelial dysfunction and coagulation activation are key features of severe COVID-19. Fibrinogen and D-dimer levels are typically increased. The risk for venous thromboembolism is markedly increased, especially in patients in the intensive care unit despite prophylactic dose anticoagulation. Pulmonary microvascular thrombosis has also been described and the risk for arterial thrombotic diseases also appears to be increased while bleeding is less common than thrombosis, but it can occur. Evaluation for venous thromboembolism may be challenging because symptoms of pulmonary embolism overlap with COVID-19, and imaging studies may not be feasible in all cases. The threshold for evaluation or diagnosis of thromboembolism should be low given the high frequency of these events. Management and treatment are new challenges due to the paucity of high-quality evidence regarding efficacy and safety of different approaches to prevent or treat thromboembolic complications of the disease. All inpatients should receive thromboprophylaxis unless contraindicated. Some institutional protocols provide more aggressive anticoagulation with intermediate or even therapeutic dose anticoagulation for COVID-19 patients admitted to ICU. Therapeutic dose anticoagulation is always appropriate to treat deep venous thrombosis or pulmonary embolism, unless contraindicated. This article reviews evaluation and management of coagulation abnormalities in individuals with COVID-19.

## 1. Introduction

A novel coronavirus was identified in late 2019 that rapidly reached pandemic proportions. The World Health Organization has designated the disease caused by the virus (severe acute respiratory syndrome coronavirus 2 (SARS-CoV-2)) as coronavirus disease 2019 (COVID-19) [1,2,3].

Bilateral pneumonia, acute respiratory failure (ARF), systemic inflammation, endothelial dysfunction and coagulation activation are key features of severe COVID-19 [4,5,6].

An increased risk of venous thromboembolism (VTE) in patients with COVID-19 pneumonia admitted to intensive care unit (ICU) [7,8,9] and in non-ICU wards has been reported despite adequate thromboprophylaxis [10,11].

Thus, several authors suggested that higher anticoagulation targets that are unusual in critically ill patients, should probably be taken into consideration for patients with COVID-19 pneumonia.

This review provides practical information for evaluation and management of coagulation abnormalities in individuals with COVID-19 [12,13].

## 2. SARS-COV-2 Clinical Feature

SARS-CoV-2 outcome seems to be determined by the extent of the host immune system imbalance. The primary immune response usually leads to viral clearance. However, for unclear reasons, the secondary immune response may be exaggerated and, in some cases, leads to multiple organ failure, acute respiratory distress syndrome (ARDS) and death [14].

This exaggerated response is known as cytokine release syndrome (CRS), and it has an important role in the activation of coagulation.

The spectrum of symptomatic COVID-19 infection ranges from mild to critical; most infections are not severe.

Specifically, in a report from the Chinese Center for Disease Control and Prevention that included approximately 44,500 confirmed infections, it was reported that 81% had a mild severity disease (without or mild pneumonia). Severe disease (e.g., dyspnea, hypoxia, or >50% lung involvement on imaging within 24 to 48 h) was described in 14%. Critical disease (e.g., with respiratory failure, shock, or multiorgan dysfunction) was identified in 5%. The overall case fatality rate was 2.3 percent; no deaths were reported among non-critical cases. Among hospitalized patients, the proportion of critical or fatal disease is higher [15].

In a study that included 2741 patients which were hospitalized for COVID-19 in a New York City health care system, 665 patients (24%) died or were discharged to hospice.

Of the 749 patients that received intensive care (27% of the total hospitalized cohort), 647 received invasive mechanical ventilation; of these patients, 60% died and 13% remained on ventilation [16].

In Italy, the rate of ICU admission was about 12% for all detected COVID-19 cases and 16% for all hospitalized patients and the estimated case fatality rate was 7.2 percent [17].

Individuals of any age can acquire SARS-CoV-2 infection, although youngest-old, middle-old and oldest-old are most commonly affected, and middle-old and oldest-old are more likely to have severe disease. Symptomatic infection in children and adolescents appears to be relatively rare; when it occurs, it is usually mild, although a small proportion (e.g., <2 percent) experience severe and even fatal disease. Severe illness can manifest in healthy individuals, but it predominantly occurs in adults with underlying medical comorbidities. Comorbidities and other conditions that have been associated with severe illness and mortality include: cardiovascular disease, diabetes mellitus, hypertension, chronic lung disease, cancer (in particular, haematological malignancies, lung cancer, and metastatic disease), chronic kidney disease, obesity and smoking. In a report of 355 patients who died with COVID-19 in Italy, the mean number of pre-existing comorbidities was 2.7, and only three patients had no underlying condition [18].

Asymptomatic infections have also been well documented [19] and the proportion of infections that are asymptomatic has not been systematically and prospectively studied. One literature review estimated that it is as high as 30 to 40 percent, based on data from two large cohorts that identified cases through population-based testing [20].

The incubation period for COVID-19 is generally within 14 days following exposure, with most cases occurring approximately four to five days after exposure [21].

Pneumonia appears to be the most frequent serious manifestation of infection, characterized primarily by fever, cough, dyspnea, and bilateral infiltrates on chest imaging [22].

However, other features, including upper respiratory tract symptoms, myalgias, diarrhea, and smell or taste disorders are also common. Although some clinical features (in particular smell or taste disorders) are more common with COVID-19 than with other viral respiratory infections, there are no specific symptoms or signs that can reliably distinguish COVID-19. Some patients with initially non severe symptoms may progress over the course of a week [23].

Common laboratory findings among hospitalized patients with COVID-19 include lymphopenia, elevated aminotransaminase levels, elevated lactate dehydrogenase levels, elevated inflammatory markers (e.g., ferritin, C-reactive protein, and erythrocyte sedimentation rate), and abnormalities in D-dimer levels (see Table 1).

Possible thresholds are extrapolated from published cohort data and individualized to the reference values used at our laboratory.

However, the specific thresholds are not well established. Several laboratory features, including high D-dimer levels and more severe lymphopenia, have been associated with critical illness or mortality [24].

Data emerging from the studies so far conducted indicate that a high value of high-sensitivity troponin represents a negative prognostic indicator when associated with heart damage on an infectious-inflammatory basis (i.e., myopericarditis) [24].

The most common abnormal chest x-ray findings were consolidation or ground-glass opacities, with a bilateral and/or peripheral and/or lower lung zone distribution, while for chest CT, the abnormalities seen ground-glass opacification with or without consolidative abnormalities and the distribution is like that seen on the chest x-ray. Among patients who improve clinically, resolution of radiographic abnormalities may result after improvements in fever and hypoxia [25].

Several complications of COVID-19 have been described. ARDS is the major complication in patients with severe disease. Other principal complications like arrhythmias, acute cardiac injury, and shock as well as thromboembolic complications, including pulmonary embolism and acute stroke have also been reported. Inflammatory complications and auto-antibody-mediated manifestations have been described: Guillain-Barré syndrome may occur, with onset 5 to 10 days after initial symptoms and multisystem inflammatory syndrome with clinical features similar to those of Kawasaki disease and toxic shock syndrome has also been described in children with COVID-19 [26].

The proportion of patients with COVID-19 who are diagnosed with ARDS on the basis of oxygenation criteria ranges between 20% and 67% in patients admitted to hospital and is 100% in mechanically ventilated patients [25,26].

Recently, Grasselli et al. published a systematic analysis of clinical and laboratory features in patients with COVID-19-associated ARDS in 301 consecutive patients prospectively enrolled in seven Italian hospitals. They compared the pathophysiology of ARDS related to COVID-19 with classical ARDS using two large historical datasets, showing that patients with ARDS-COVID-19 have an injury pattern that is similar to that of classical ARDS characterized by decreased compliance and increased lung weight. In many patients, this injury was complicated by increased dead space which was probably related to diffuse microthrombi or emboli of the pulmonary vasculature.

In this study, patients with ARDS related to COVID-19 had a median static compliance of the respiratory system 28% higher (*n* = 297; 41 mL/cm H_2_O [IQR 33–52]) in those with no COVID-19 ARDS related (*n* = 960; 32 mL/cm H_2_O [25], *p* < 0.0001). Moreover, they found that most of the patients had markedly increased D-dimer concentrations (median 1880 ng/mL [IQR 820–6243]).

In this study, 28-day mortality was 36% (93 of 261 patients). In particular, when an easily identified phenotype of increased parenchymal damage (low static compliance) and increased D-dimer concentrations occurs together, mortality is extremely high [27].

Secondary infections including respiratory infections and bacteremia do not appear to be common complications of COVID-19 overall, although data are limited [28].

Several reports have described presumptive invasive aspergillosis among immunocompetent patients with COVID-19-ARDS, although the frequency of this complication is uncertain. [29]

Recovery time appears to be around two weeks for mild infections and three to six weeks for severe disease based on early data from China. However, the recovery course is variable and depends on age and pre-existing comorbidities, in addition to illness severity. Systematic evaluation of the long-term sequelae of COVID-19 suggest a likely chronic respiratory impairment. Moreover, cardiac imaging studies have also suggested cardiac sequelae after COVID-19 [30,31].

## 3. Coagulation Abnormalities in Patients with COVID-19

SARS-CoV-2 may predispose patients to thrombotic disease, both in the venous and arterial circulation, due to excessive inflammation, platelet activation, and endothelial dysfunction [32,33,34,35].

Bleeding does not appear to be a major manifestation of COVID-19, but patients may have bleeding for other reasons, including trauma and/or treatment with anticoagulation. If it occurs, treatment is similar to non-COVID-19 patients and may include transfusions, anticoagulant reversal or discontinuation, or hemoderivates for underlying bleeding disorders.

Laboratory findings in COVID-19 (see Table 2) are: prothrombin time (PT) and activated partial thromboplastin time (aPTT) normal or slightly prolonged, platelet counts normal or increased, fibrinogen increased, D-dimer increased, factor VIII activity increased, von Willebrand factor (VWF) antigen greatly increased, minor changes in natural anticoagulants (i.e., slight decreases in antithrombin and mild increase in protein C) [33,34,35,36].

The presence of a lupus anticoagulant (LA) is common in individuals with a prolonged aPTT.

Very elevated levels of D-dimer have been observed that correlate with illness severity, especially if levels are increased several-fold [37].

This state appears to be distinct from disseminated intravascular coagulation (DIC), even if some critical patients with COVID-19 have met criteria for probable DIC.

Clinical findings of acute DIC include bleeding, thrombocytopenia, prolonged PT and aPTT, low plasma fibrinogen, elevated plasma D-dimers, and microangiopathic changes on the peripheral blood smear. The International Society of Thrombosis and Haemostasis (ISTH) has developed in 2009 a scoring system to be applied to individuals with an underlying disorder associated with DIC, which incorporates laboratory features including the PT, platelet count, fibrinogen level, and D-dimer [38].

The ISTH scoring system (see Table 3) is reported to have a sensitivity of 91% and a specificity of 97%, but is not widely used.

COVID-19 has similar laboratory findings to DIC, including a marked increase in D-dimer and in some cases, mild thrombocytopenia, but other coagulation parameters in COVID-19 are distinct from DIC. In particular, the typical findings of elevated fibrinogen and factor VIII activity suggest that major consumption of coagulation factors is not occurring. [39]

In one of the largest series that reported on thromboembolic events related to COVID-19, none of the patients developed overt DIC. [40]

The pathogenesis of hypercoagulability in COVID-19 is not completely understood, however, we know that cytokine release syndrome (CRS) has an important role to play in the pathogenesis and disease severity [41].

CRS is associated with increased levels of inflammatory cytokines and activation of T lymphocytes, macrophages, and endothelial cells. In particular, interleukin 6 and tumor necrosis factor seems to hold a key role leading to vascular leakage and to activation of complement, tissue factor and coagulation cascade [42,43].

Moreover, endothelial injury, stasis and a hypercoagulable state known as Virchow’s triad are present for the clot formation in the severe COVID-19 infection. There is evidence of direct invasion of endothelial cells by the severe acute respiratory syndrome coronavirus 2 (SARS-CoV-2) virus, potentially leading to endothelial injury [44].

Other sources of endothelial injury include intravascular catheters and mediators of the acute systemic inflammatory response such as cytokines (e.g., interleukin [IL]-6) and other acute phase reactants and the contribution of complement-mediated endothelial injury has also been suggested [45,46].

Immobilization can cause stasis of blood flow in all hospitalized and critically ill patients, regardless of having COVID-19; but little changes in circulating prothrombotic factors have been found in patients with severe COVID-19: elevated factor VIII, elevated fibrinogen, neutrophil extracellular traps (NETs) and hyperviscosity [47,48].

The hypercoagulable state has a dominant role to play together with the Acute Respiratory Failure and the principal question is: prevention or treatment of thrombosis [49]?

## 4. Venous Thromboembolism in Critically Ill Patients

Critically ill patients have an increased risk of VTE of the upper and lower extremities. The risk factors include immobility associated with serious illness such as sepsis and trauma, and invasive procedures such as central venous lines [50].

The most serious manifestation of VTE is pulmonary embolism (PE). Of all PEs, 90% are estimated to originate from deep venous thrombosis (DVT) of the lower limbs. DVT and PE share common risk factors [51,52].

The main clinical importance of DVT lies in its association with potentially life-threatening PE. In critically ill patients with impaired cardiopulmonary reserve, a mild PE might have severe or fatal sequelae. In addition, evaluation for VTE in critical ill patients may be challenging. Thus, some mechanically ventilated patients with sudden episodes of hypotension, tachycardia, or hypoxia may have undetected PE [53].

PE is stratified into massive, sub-massive, and low-risk based upon the presence or absence of hypotension and right ventricular dysfunction or dilation: this stratification is associated with mortality risk [54].

The prevalence of VTE in non-COVID-19 ICU patients ranged from 2 to 8% [55].

In a retrospective observational cohort study in 12 adult ICUs, including 12.338 medical-surgical critically ill patients, VTE appears to be an apparently infrequent problem, occurring also among patients receiving prophylaxis [56]. Indeed, only 1–2% of patients developed VTE. Across these 12 ICUs, the incidence of definite DVT or PE ranged from 0.1–2.6% and 0.2–2.4%, respectively. In particular, 252 (2.0%) patients had confirmed VTE (166 DVT events and 122 PE events). Most incident events occurred within 2 weeks of ICU admission. Two thirds of patients required mechanical ventilation and one third required vasopressors or inotropes during their ICU stay. The proportion of patients with VTE who received thromboprophylaxis for 80% or more of their ICU stay was 65.8%. Thus, most VTE events were due to prophylaxis failure rather than failure to provide prophylaxis [56].

## 5. Venous Thromboembolism in Hospitalized Patients with COVID-19 Pneumonia

Among hospitalized COVID-19 patients, an increased risk of VTE has been reported despite adequate thromboprophylaxis [57,58].

A higher prevalence of VTE was found, in comparison to non-COVID-19 ICU patients (2–8%).

Moreover, COVID-19 patients in the ICU had a higher risk of VTE than those in the ward.

Case series of ICU patients including more than 600 patients reported high rates of VTE (range 20–43%), mostly PE. Data regarding VTE rate outside the ICU are more limited, but also suggest a possibly increased rate (range 3–6%). Other studies that focused on COVID-19 patients also show a higher rate of DVT (65–69% in ICU patients [57,58].

**Figure 1:**
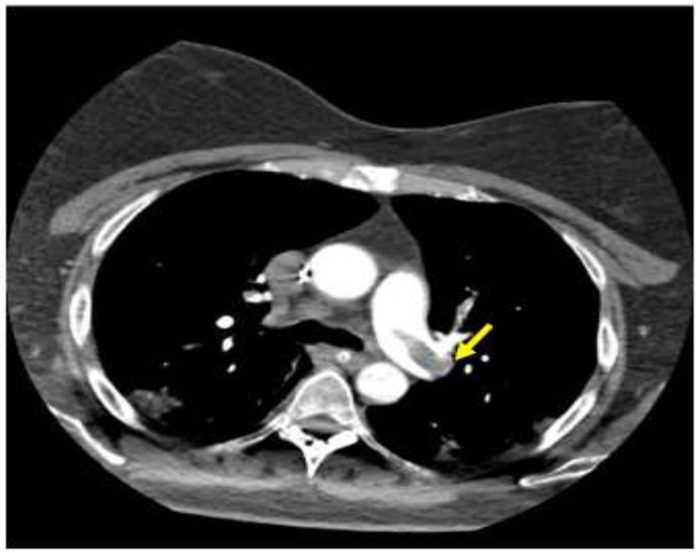
Computed tomography scan imaging of acute respiratory distress syndrome (ARDS) associated with COVID-19 and pulmonary emboli: pulmonary embolus across the bifurcation of the pulmonary trunk is noted, as indicated by the arrow.

In a retrospective study of 62 patients with ARDS related to COVID-19, 11 diagnoses of PE were described and in all of these patients, the main pulmonary arteries were involved (Figure 1).

**Figure 2:**
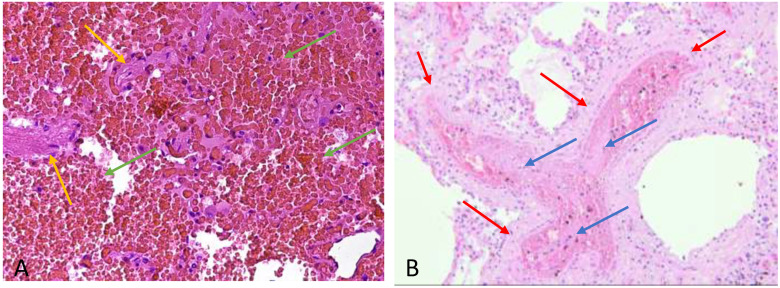
(**A**) Extensive intra-alveolar hemorrhage, the alveolar spaces (alveolar septum yellow arrow) are filled by erythrocyte (green arrow) without fibrin and granulocytes (H&E 200 X) (**B**) Microvascular thrombi (H&E 100 X) in the arteriolar lumen (arteriole wall red arrow) there is microembolism consisting of fibrinous meshes that retain leukocytes and platelets (blue arrow).

Autopsy studies in small series of patients who have died from COVID-19 have also demonstrated microvascular thrombosis and intra-alveolar blood extravasation in the lungs (Figure 2) [57,58,59].

Before the autopsy CT post mortem can be performed to identify the cause of death [60].

The universality and clinical implications of these observations require further research.

Several factors contribute to the increase of VTE risk in ICU patients. Recognized risk factors for DVT are related to one or more elements of Virchow’s triad: flow stasis, vessel injury and hypercoagulability. Flow stasis, due to prolonged immobility, mechanical ventilation, use of sedatives and neuromuscular blocking drugs, play a major role in ICU patients.

In addition, in this population vessel injury may be due to catheter insertion in central veins and hypercoagulability may be induced by sepsis or dehydration [9].

Evaluation for DVT or PE in these patients may be challenging because symptoms of PE overlap with COVID-19, and imaging studies may not be feasible in all cases [9,10,57].

The threshold for evaluation or diagnosis of DVT or PE should be low given the high frequency of these events and the presence of additional VTE risk factors in many individuals. In patients with suspected PE due to unexplained hypotension, tachycardia, worsening respiratory status, or other risk factors for thrombosis, computed tomography with pulmonary angiography is the preferred test to confirm or exclude the diagnosis. On the other hand, bilateral complete duplex ultrasound (CDUS) is the suggested test to screen for DVT.

Heparin resistance (requirement for very high doses of heparin to achieve a therapeutic aPTT or anti-factor Xa activity) might be another concern in acutely ill patients with COVID-19. In a French study, 43% of patients reported VTE despite thromboprophylaxis, and thrombotic complications occurred despite prophylactic or therapeutic anticoagulation, respectively, in 70% and 30% of patients. In another series, among 74 patients, VTE was reported in 29 patients [9].

All of them were receiving anticoagulation, both at prophylactic and therapeutic levels. In addition, a series of 15 individuals in the ICU anticoagulated for VTE noted a very high requirement for unfractionated heparin or low molecular weight heparin. In particular, five of five receiving dalteparin had anti-factor Xa peak below expected (<0.6 international units/mL for twice daily dosing or <1 international units/mL for once daily dosing) [61].

The reason for heparin resistance is not understood; the authors stated that heparin is negatively charged and can interact with a variety of positively charged plasma proteins, some of which behave like acute phase reactants and will compete for heparin binding. Furthermore, the suboptimal efficacy of higher anticoagulation dose could also be explained by the underlying pathophysiological mechanism which explains the presence of thrombotic material in pulmonary circulation. In the context of COVID-19, pulmonary thrombosis may develop via a distinctive mechanism, and therefore may not respond adequately to intensified anticoagulation [9,10].

Based on these reports, many physicians are advocating the empiric use of therapeutic anticoagulation even in patients who do not have a documented diagnosis of VTE. On the other hand, the current position of the majority of medical societies still recommend using standard prophylactic doses of anticoagulation for hospitalized COVID-19 patients, similar to those that are recommended for other acutely ill medical patients.

In one north eastern ICU, a high prevalence of PE was registered among the first 62 patients (19.3% cases) affected by COVID-19-related ARF, admitted from 1–31 March 2020, despite regular antithrombotic prophylaxis [9,10].

Thus, a protocol with increased doses of thromboprophylaxis was introduced in this hospital for these patients. Subsequently, the same hospital performed a prospective, observational study to assess thrombotic risk in COVID-19 pneumonia patients and to compare populations treated with three different antithrombotic prophylaxis protocols [9,10].

Seventy-four patients were enrolled (44 men and 30 women, average age 68.6). Diagnosis of venous thromboembolism was made in 21 cases (28.4%). Forty-seven out of 74 patients (63.5%) received intermediate or therapeutic dose of anticoagulation, while twenty-seven patients (34.5%) received standard antithrombotic prophylaxis. The analysis showed that an intermediate or therapeutic dose of anticoagulation did not decrease the prevalence of thrombotic events. On the other hand, six patients reported severe hemorrhagic complications (two cases in the standard antithrombotic prophylaxis group and four cases with increased antithrombotic dose), with hemorrhagic shock in three cases. In addition, mortality among patients receiving a higher dose of antithrombotic prophylaxis was three times higher than in subjects treated with standard prophylaxis. Given the limited total number of patients in the Longhitano’s study and the non-randomized design, it may be premature to disprove the benefit of higher dose thromboprophylaxis. Besides, a small randomized trial (HESACOVID) randomly assigned 20 individuals with severe COVID-19 to receive therapeutic dose anticoagulation (enoxaparin, 1 mg/kg twice daily) or prophylactic dose anticoagulation (enoxaparin, 40 mg once daily or unfractionated heparin, 5000 units three times daily); adjustments were made for age, weight, and kidney function as appropriate [57,58,59,60,61,62].

Half the patients in the prophylactic group received unfractionated heparin and half enoxaparin. Compared with prophylactic dosing, therapeutic dosing led to fewer days on the ventilator and significant reductions in D-dimer levels. Even for this study, confidence in the results is hampered by the open-label design and small size.

Consequently, data comparing different levels of anticoagulation (prophylactic, intermediate, or therapeutic dosing) in severely ill or critically ill patients are extremely limited, and the choice between different anticoagulation treatments for thromboprophylaxis is a challenge. Hypercoagulability appears to adversely impact on prognosis, but there are no high-quality studies to support interventions that go beyond standard indications, while antithrombotic therapies increase the risk of bleeding. In addition, clinical trials aiming to determine the best approach for critically ill patients are in progress.

To date, VTE prophylaxis using at least prophylactic dosing is appropriate in all hospitalized medical, surgical, and obstetric patients with COVID-19, unless there is a contraindication to anticoagulation (e.g, active bleeding or serious bleeding in the prior 24 to 48 h) [63].

Some institutional protocols provide more aggressive anticoagulation with intermediate or even therapeutic dose anticoagulation for COVID-19 patients admitted to ICU. Low molecular weight (LMW) heparin is preferred for thromboprophylaxis, but unfractionated heparin can be used if LMW heparin is unavailable or if kidney function is severely impaired. In case of history of heparin-induced thrombocytopenia (HIT), an alternative agent such as fondaparinux may be used. The presence of a prolonged aPTT due to the lupus anticoagulant (LA) phenomenon does not reflect decreased risk of thromboembolic complications (in some individuals, it reflects increased risk) and is not a reason to avoid anticoagulation. On the other hand, therapeutic dose (full dose) anticoagulation for at least three months is always appropriate to treat DVT or PE, and tissue plasminogen activator (tPA) is appropriate for massive PE, unless there is a contraindication.

## 6. Arterial Thrombosis

There are also reports of arterial thrombosis, including in the central nervous system. The largest study, which included 3334 individuals (829 ICU and 2505 non-ICU) reported stroke in 1.6 percent and myocardial infarction in 8.9 percent [64].

Risk factors for arterial thrombosis included older age, male gender, Hispanic ethnicity, history of coronary artery disease, and D-dimers > 230 ng/mL on presentation. Arterial thrombotic events were associated with increased mortality.

A report described 20 patients with COVID-19 who developed acute limb ischemia at a single institution over a three-month period [65].

This represented a significant increase in limb ischemia over the previous year (16 percent, versus 2 percent in early 2019). Most were male, and the average age was 75 years. Surgical revascularization procedures were performed in 17, of which 12 (71 percent) were successful, a lower-than-expected success rate. Individuals who received postoperative heparin did not require reintervention, although the benefits of postoperative heparin did not reach statistical significance.

## 7. Immuno-Thrombosis and Anticoagulation Therapy

Infection of endothelial cells by severe acute respiratory syndrome coronavirus 2 (SARS-CoV-2) and liberation of viral danger-associated molecular pattern (DAMPs) results in endothelial activation with the upregulation of tissue factor (TF) and adhesion molecule expression in addition to endothelial cytokine production. This leads to the recruitment and activation of leukocytes and platelets which release neutrophil extracellular traps (NETs), monocyte-derived, TF-bearing microvesicles (MV), polyP [66].

Thrombin exerts its thrombotic effect by activating platelets through the platelet PAR (protease-activated receptor), in addition to mediating the cleavage of fibrinogen to fibrin. Furthermore, thrombin possesses proinflammatory functions due to its ability to activate endothelial cells and leukocytes. Thrombin activates endothelial cells, leading to upregulation of IL-6, IL-8, PAF (platelet-activating factor), and MCP (monocyte chemoattractant protein)-1 in addition to the adhesion molecules P-selectin, E-selectin and ICAM (intercellular adhesion molecule)-1, all of which serve to increase leukocyte recruitment and activation [66].

The resultant endothelial cell, platelet, and leukocyte interactions establish a positive feedback loop, which further promulgates ongoing thrombin generation leading to immunothrombosis. The clinical manifestations of this proposed role of thrombin seen in COVID-19, include: pulmonary embolism (PE), microvascular thrombosis, ischemic stroke, and deep vein thrombosis (DVT) [66].

*Heparins.* Preliminary reports have suggested that low molecular weight heparin treatment reduces mortality in COVID-19 patients with an elevated D-dimer or elevated sepsis-induced coagulopathy score [67].

The heparins are widely used anticoagulants which inhibit coagulation by the antithrombin mediated inhibition of FXa or thrombin and they have pleiotropic effects that may provide unique advantages in the context of viral infection, including anti-inflammatory effects by way of their ability to bind to danger associated molecular patterns, such as HMGB-1, and proinflammatory cytokines [68,69].

Recent studies have directly investigated the link between heparin and SARS-CoV 2 and suggest that heparin can directly bind with the spike protein of the SARS-CoV-2 virus and, therefore, may have antiviral properties.146. It is important to note that given the potential for heparin therapeutics to cause heparin-induced thrombocytopenia, but reassuringly, despite both thrombocytopenia and thrombosis being common complications of COVID-19, the development of heparin-induced thrombocytopenia appears limited to individual case reports [70].

*Fibrinolytics.* tPA (tissue-type plasminogen activator) converts the inactive enzyme plasminogen to plasmin leading to the subsequent breakdown of cross-linked fibrin. The interest in tPA has emerged from the concept that ARDS is characterized by significant local inflammatory reaction in addition to a hypofibrinolytic state.

The use of systemic tPA in preclinical and human models of ARDS demonstrates improved oxygenation, and a case report of tPA use in severe COVID-19 showed an initial improvement in oxygenation during tPA infusion, but this effect was lost after tPA therapy ceased [71,72,73].

The risk of major bleeding was seen in non-stroke clinical trials, 159 which has led to the consideration of nebulized therapy to increase local concentrations and reduce systemic coagulation effects. Recent clinical trial data suggest that, in severe ARDS, nebulized fibrinolytic therapy is associated with improved oxygenation and ventilatory parameters, which may be another plausible treatment option in COVID-19 [72].

*Antiplatelet Agents.* The majority of data linking antiplatelet therapy with these improved clinical outcomes relates to aspirin.161 prevents intravascular coagulation and neutrophil mediated microvascular thrombosis in a mouse model of bacterial sepsis.162, while in the COVID-19 the aspirin effects remain to be established.

Dipyridamole has recently been reported to suppress SARS-CoV-2 replication in vitro, with some early data suggesting that use of adjunct dipyridamole may improve the clinical course in severe COVID-19 [74].

Nafamostat, a serine protease inhibitor which has antiplatelet effects and is currently marketed in Asia for the treatment of disseminated intravascular coagulation and pancreatitis. Nafamostat is known to inhibit TMPRSS-2, and in preclinical studies, serine protease inhibitors are able to block SARS-CoV-2 infection of lung cells. Prior studies of nafamostat demonstrated that the drug has potential in blocking MERS-CoV infection in vitro [66].

*Direct Oral Anticoagulant.* Ongoing DOAC use was not associated with reduced risk of severe COVID-19, indicating that prognosis would not be modified by early outpatient DOAC initiation [75].

In one small study DOAC patients treated with antiviral drugs show an alarming increase in DOAC plasma levels. In order to prevent bleeding complications, we believe that physicians should consider withholding DOACs from patients with SARS-CoV-2 and replacing them with alternative parenteral antithrombotic strategies for as long as antiviral agents are deemed necessary and until discharge.

*Cortiscosteroids.* In 2006, a meta-analysis on the use of steroids in patients with SARS. Among the 29 evaluated studies, 25 were inconclusive and four concluded that steroids should not be used in SARS-CoV-1 infection, since they were associated with increased mortality. Additionally, high dose of steroid therapy was associated with diabetes and with SARS-related psychosis. A study of 30 patients with SARS-CoV-1 infection treated with methylprednisolone showed that the initial stage of the disease is characterized by a reduction of CD4^+^, CD8^+^, and CD3^+^ cells and, therefore, immunosuppression can be worsened by the administration of high-dose steroids, increasing the risk of serious secondary infections.

Despite of dearth of previous conclusive studies on steroid therapy in COVID-19 and the steadily increasing number of infected patients, the Chinese Thoracic Society (CTS) released an expert consensus on the use of steroids, declaring that: (i) the benefits and risks must be carefully weighed before using steroids; (ii) steroids should be used with caution in critically ill patients; (iii) greater caution should be paid for patients with hypoxemia due to underlying diseases or who regularly use steroids for chronic diseases; and (iv) the dose administered should be low to moderate (≤0.5−1 mg/kg/day methylprednisolone or equivalent), and the duration short (≤7 days). Additionally, CTS experts recommend against the indiscriminate use of steroids in COVID-19 [76].

Finally, steroids are generally safe drugs in short-term use, despite the potential for adverse effects such as temporary hyperglycemia. Prolonged use, however, may be associated with adverse events such as glaucoma, cataracts, fluid retention, hypertension, psychological effects, weight gain, or increased risk of infections and osteoporosis. Additionally, clinicians should be aware of possible drug–drug interactions, since dexamethasone is a moderate inducer of cytochrome P450 (CYP)3A4, and may thus impact the concentration and effects of other medications that might be CYP3A4 substrates [77,78,79].

*Extra Corporeal Membrane Oxigenation.* In patients with COVID-19 supported with ECMO, both estimated mortality 90 days after ECMO initiation and mortality in those who achieved a final disposition of death or discharge were less than 40%. The results were similar when the sample was limited to patients with COVID-19 who were characterised as having ARDS. Our results are also consistent with previously reported survival rates in acute hypoxaemic respiratory failure, supporting current recommendations that centres experienced in ECMO should consider its use in refractory COVID-19-related respiratory failure [13,80].

## 8. Conclusions

COVID-19 is characterized by a hypercoagulable state associated with acute inflammatory changes and laboratory findings that are distinct from DIC, except for some patients with very severe disease. Fibrinogen and D-dimers are typically increased. The pathogenesis of these abnormalities is incompletely understood, and there may be many contributing factors related to the acute inflammatory response to the disease.

The risk for VTE is markedly increased, especially in patients in the ICU, often despite prophylactic dose anticoagulation. Pulmonary microvascular thrombosis has been also described, and the risk for arterial thrombotic events such as stroke, myocardial infarction, and limb ischemia also appears to be increased. Bleeding is less common than thrombosis but can occur.

Evaluation for VTE may be challenging because symptoms of PE overlap with COVID-19, and imaging studies may not be feasible in all cases, but the bedside Point of Care of Ultrasound remains primarily an excellent tool to start the evaluation and subsequent management and treatment [81].

**Figure 3:**
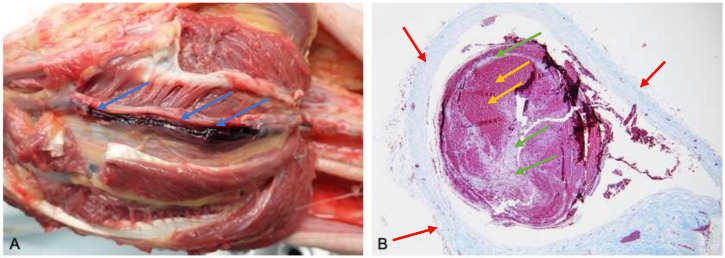
(**A**) Macroscopic autopsy findings. Thrombi are noted in the lumen of the posterior tibial vein (blue arrow). (**B**) Histological examination (Masson’s trichrome 60 X), the antemortem thrombosis within the vein lumen (vein wall red arrow), which contain the so-called “lines of Zahn” (green arrow), representing the layerwise, alternating deposition of erythrocytes (yellow arrow), leukocytes and fibrin within the vessel lumen.

Most often the diagnosis of VTE is made during an autopsy and confirmed by histological examination (Figure 3).

The threshold for evaluation or diagnosis of DVT or PE should be low given the high frequency of these events and the presence of additional VTE risk factors in many individuals. In patients with suspected PE, computed tomography with pulmonary angiography is the preferred test to confirm or exclude the diagnosis. CDUS is the suggested test to screen for DVT.

Management is challenging due to the paucity of high-quality evidence regarding efficacy and safety of different approaches to prevent or treat thromboembolic complications of the disease. All inpatients should receive thromboprophylaxis unless contraindicated. Some institutional protocols provide more aggressive anticoagulation with intermediate or even therapeutic dose anticoagulation for COVID-19 patients admitted to ICU. Therapeutic dose anticoagulation is always appropriate to treat DVT or PE, unless contraindicated.

Finally, another hopeful drug is Factor XII inhibitor that can inhibit the thrombo-inflammatory response incited by severe COVID-19. In animal models, this inhibitor demonstrates protection from occlusive thrombus formation without impeding hemostasis. Importantly, the potential safety of FXII inhibitors is highlighted by the fact that FXIIdeficient individuals display no bleeding phenotype, and there is no known effect on immune function [66,82,83].

## Figures and Tables

**Table 1:** Laboratory features associated with severe COVID-19.

	Possible Threshold
**↑** **D-Dimer**	>1000 ng/mL (normal range: <500 ng/mL)
**↑** **C-Reactive Protein**	>100 mg/L (normal range: <8.0 mg/L)
**↑** **Lactate Dehydrogenase**	>245 units/L (normal range: 110 to 210 units/L)
**↑** **Ferritin**	>500 mcg/L (normal range: females 10 to 200 mcg/L; males 30 to 300 mcg/L)
**↑** **Troponin**	>2 × the upper limit of normal
**↑ Creatine Phosphokinase**	>2 × the upper limit of normal
**↓ Absolute Lymphocyte Count**	<800/microL (normal range for age ≥ 21 years: 1800 to 7700/microL)

**Table 2:** The hypercoagulable state and predominant coagulation abnormalities in patients with covid-19 [Panigada M, Bottino N, Tagliabue P, et al. Hypercoagulability of COVID-19 patients in Intensive Care Unit. A Report of Thromboelastography Findings and other Parameters of Hemostasis. J Thromb Haemost 2020].

✓ **D-dimer increased** ✓ **fibrinogen increased** ✓ prothrombin time (PT) and aPTT * normal or slightly prolonged ✓ **platelet counts normal or increased** ✓ **factor VIII activity increased** ✓ **VWF antigen greatly increased** ✓ **small decreases in antithrombin and small increases in protein C**

* The presence of a LA is common in individuals with a prolonged aPTT.

**Table 3:** The International Society on Thrombosis and Haemostasis (ISTH) scoring system. A ISTH score of 5 or more points suggests DIC is probable.

✓ **thrombocytopenia:** o 1 point for platelet count 50,000 to 100,000/microL o 2 points for platelet count r < 50,000/microL ✓ **prolonged PT:** o 1 point for 3 to 6 s of prolongation o 2 points for more than 6 s of prolongation ✓ **low fibrinogen: 1 point for <100 mg/dL** ✓ **increased D-dimer:** o 2 points for moderate increase o 3 points for “strong” increase

## Data Availability

We choose to exclude this statement.
